# Heater-Integrated Cantilevers for Nano-Samples Thermogravimetric Analysis

**DOI:** 10.3390/s131216657

**Published:** 2013-12-04

**Authors:** Valeria Toffoli, Sergio Carrato, Dongkyu Lee, Sangmin Jeon, Marco Lazzarino

**Affiliations:** 1 IOM-CNR Laboratorio TASC, SS 14 km 163, 5, Trieste 34012, Italy; E-Mail: toffoli@iom.cnr.it; 2 DIA-Università di Trieste, Via Valerio, Trieste 10-34127, Italy; E-Mail: carrato@univ.trieste.it; 3 University of Alberta, 116 St and 85 Ave, Edmonton, Alberta T6G 2R3, Canada; E-Mail: dongkyu@ualberta.ca; 4 Department of Chemical Engineering, Pohang University of Science and Technology, Pohang 790-784, Korea; E-Mail: jeons@postech.ac.kr

**Keywords:** microelectromechanical system (MEMS) resonator, mass sensing, thermogravimetric analysis (TGA), microcapsules

## Abstract

The design and characteristics of a micro-system for thermogravimetric analysis (TGA) in which heater, temperature sensor and mass sensor are integrated into a single device are presented. The system consists of a suspended cantilever that incorporates a microfabricated resistor, used as both heater and thermometer. A three-dimensional finite element analysis was used to define the structure parameters. TGA sensors were fabricated by standard microlithographic techniques and tested using milli-Q water and polyurethane microcapsule. The results demonstrated that our approach provides a faster and more sensitive TGA with respect to commercial systems.

## 1. Introduction

The use of thermal analysis is nowadays widespread in pharmaceutical, university and research laboratories [1,2]. Thermal analysis, in standard applications, requires sample masses in the milligram range [3–5] and is usually classified into two main groups: gravimetric analysis (TGA) and differential scanning calorimetry (DSC). In particular, TGA involves the detection of the mass change caused by a temperature scan: a material loses weight with temperature, because of evaporation and/or sublimation, or increases weight in reacting environments, e.g., in oxidative processes. The obtained mass-temperature curve is typical for each material and can be used as a benchmark for synthesis characterization. The main drawback of commercially available TGA systems is generally due to a poor mass sensitivity, which results in destruction of gram quantities of the compound under investigation and poses serious problems for expensive and rare materials. The purpose of this work is to use MEMS technologies to develop a micromechanical sensor that allows one to reduce the quantity of sample required in typical TGA analysis [6]. Indeed, micro-cantilevers offer high sensitivity as mass sensors [7], fast thermal response [8] thanks to their reduced size and may be used to detect the variation in adsorbed material with single molecule sensitivity [9]. Moreover silicon micro-cantilevers are compatible with silicon micro-fabrication processes, therefore they can be integrated with silicon microelectronic circuits and can be produced on large scale at low price [10].

Berger *et al.* have illustrated a first step toward a fully integrated MEMS TGA device in [11], where an scanning force microscopy (SFM) cantilever has been employed for thermal analysis, by using an integrated piezoresistor as heated temperature sensor and strain gauge. In this solution the measured temperature is averaged over the whole device temperature and does not correctly represent that of the sample under test. Moreover, silicon strong dependence on temperature causes shifts on the resonance frequency of the cantilever even without a mass change of the sample under test.

In [12], a silicon cantilever is used as a sensing sensor and another as a reference sensor. An external heater is employed to warm up the entire wafer and resonance frequencies are measured with a laser Doppler vibrometer. Also in this case, a strong dependence of resonance frequency on the device temperature is shown. Further results have been presented in [13], where a cantilever equipped with a hot plate and external piezoelectric actuation were employed. Finally, in [14,15], a fully integrated MEMS TGA has been realized. The structure consists of silicon nitrite cantilever, thermal actuators, polysilicon heater and thermocouple. This configuration presents the drawback of a thermal crosstalk between the thermal actuators and the piezoresistor and, as a result, a dependence of resonance frequency on temperature.

In this paper we revisited the Berger approach, by overcoming several of its temperature limits, and implementing the paddle configuration introduced in [15]. We first describe the thermo-mechanical simulations that lead to the design of the sensor. Then we report on the fabrication of the prototypes and on the experimental set-up. Finally, devices performances obtained using Milli-Q water and chlorobenzene-containing polyurethane microcapsules are discussed.

## 2. Micro-System Design and Fabrication

### 2.1. Cantilever Design

We based our sensors on the Si_3_N_4_/silicon system for the following reasons. First of all silicon nitride layers can be produced with low residual stress, thus minimizing the bowing of the cantilever at the end of the process. The mechanical [16] and thermal [17] properties of Si_3_N_4_ do not vary significantly in the range of temperature used for our experiments; moreover Si_3_N_4_ is an excellent insulator and allows an easy integration of a joule-heating element. Finally these materials are compatible with CMOS manufacturing process.

Starting from a silicon wafer coated with 2 μm thick LPCVD silicon nitride on both sides, a cantilever composed of a narrow arm and a large square platform is designed to operate in vacuum in a frequency range from 45 to 55 kHz. A heating element is placed on one side of the platform while the sample is placed on the opposite side. For sake of simplicity in the numerical analysis, the heating element was simulated with a 100 nm thick, uniform layer of nickel, to which heat was externally provided. In real devices heat was provided through Joule effect and the heater was shaped as a zigzag meander line [18]. The frequency and thermal behaviors of the cantilever were simulated interfacing Comsol Multiphysics with a Matlab routine [19,20], to provide parametric solutions and further data elaboration.

**Figure 1.**
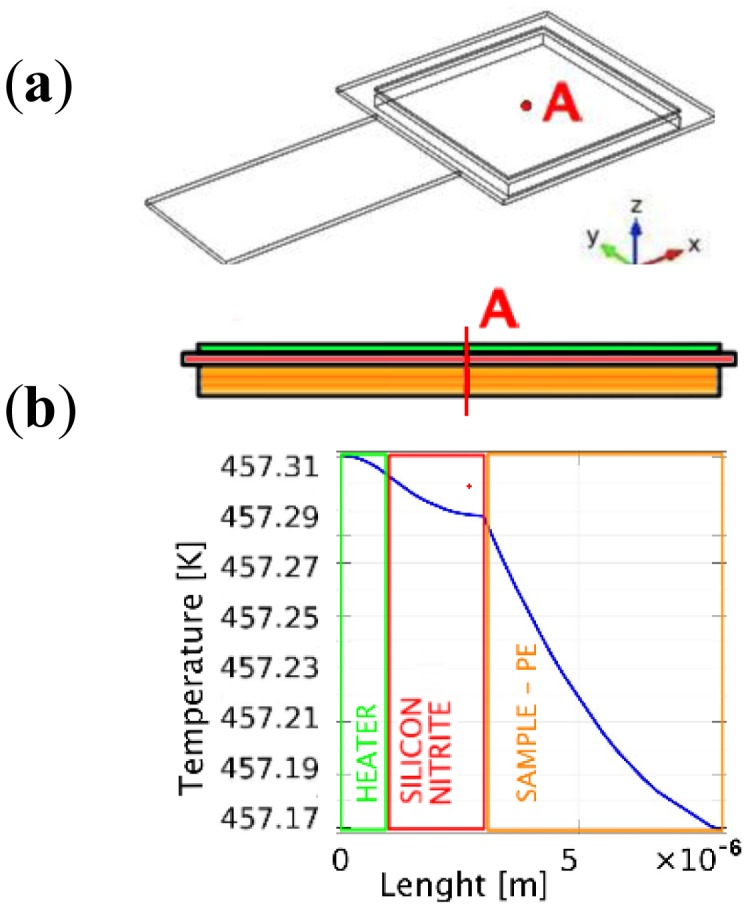
Representation of a cantilever-based TGA and temperature simulation results. (**a**) Model of the MEMS sensor realized by Comsol; (**b**) Cross-section temperature distribution on device under operation condition.

A major difference between our approach and conventional implementations of TGA systems is that in the latter the sample is placed inside a macroscopic furnace with large heat capacity, while in our system the heater is on one face of the cantilever and the sample is deposited on the opposite one. This offers the advantage of a very global small heat capacity, equal to 4.9 × 10^−8^ J/K [21], but does not guarantee uniformity. To be sure that the temperature reading on the sensor corresponds to the sample temperature, we simulated the thermal behavior of a 3 μm thick polyethylene (PE) layer deposited on the opposite side of the cantilever. The whole system was then placed in vacuum and irradiation was taken into account. PE has high thermal capacity and low thermal conductivity [22], therefore represents a good material for the evaluation of temperature dishomogeneity among those presented in COMSOL libraries. The geometry used is shown in Figure 1a (a 100 × 50 μm cantilever with, on its free end, a 100 × 100 μm paddle) and was implemented to solve the equation:

(1) $$\nabla \left(-k\nabla T\right)=Q+q_{S}T$$

where *k* is the thermal conductivity, *T* is the temperature, *q_s_* thermal absorption/production coefficient and *Q* the heat source. We performed 200 simulations by imposing a variable *Q* on the nickel platform from 10^1^ to 10^13^ W/m^3^, continuity boundary conduction, radiation from device surface to surrounding environment and a room temperature of 25 °C.

At 1,500 K the temperature remains almost unchanged across the polyethylene layer with a maximum variation of 0.045 K while it presents a gap of 0.15 K across the cantilever. The temperature distribution across the device calculated in the center of the cantilever (point A in Figure 1a) is shown in Figure 1b.

**Figure 2.**
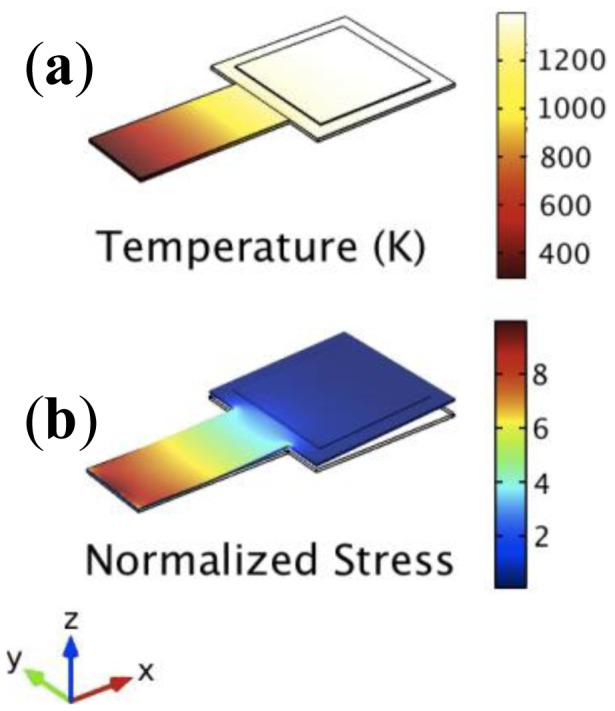
Simulation results obtain on different physical domain: (**a**) temperature distribution among the structure and (**b**) normalized displacement of the resonator at its first resonance mode. The bending stress is confined at the base of the cantilever where temperature changes are minimal, so that the influence of temperature on the elastic constant is negligible.

In order to obtain an accurate overview of the physical phenomena that took place on the resonator and how they interacts whit each other, simulations have been performed in mechanical and thermal domains separately. In particular, as shown in Figure 2, good thermal isolation of the device allows the confinement of all temperature phenomena at the cantilever platform, while areas with high stress level remain close to ambient temperature.

The resonance frequency of an unloaded cantilever is a function of its elastic modulus, density and geometry. When this structure is subjected to both uniform and local thermal phenomena, changes in these properties can affect its resonance frequency. Starting from the assumption of linear, homogeneous and isotropic materials, we performed a frequency analysis to separate the resonance frequency shift connected to the dependence of the Young module from the temperature and the resonance frequency shift induced by the variation of the sample mass. In particular, we took into account a loaded cantilever beam with two different scenarios: uniform and local temperature changes.

We first looked at a simplified structure made of a uniformed heated silicon rectangular cantilever beam with a concentrated mass (both paddle and sample) at the free end. The resonance frequency of this structure is given by [14]:

(2) $$f(T)=\frac{1}{2\pi }\sqrt{\frac{k}{m_{\text{eff}}+m_{S}}}$$

where *T* is the operating temperature, *k* = *k*(*E,t,w_1_,w_2_,l_1_,l_2_*) is the spring constant, *m_s_* = *m_s_*(*T*) is the sample mass, *m_eff_* = *m_eff_* (*t,w_1_,w_2_,l_1_,l_2_*) is cantilever effective mass, *E* = *E*(*T*) is Young's modulus, *w_2_*, *t* = *t*(*T*) and *l_2_* are cantilever width, thickness and length respectively while *w_1_* = *w_1_*(*T*) and *l_1_* = *l_1_*(*T*) are paddle width and length respectively. Sample masses *m_s_* from about 10% of the platform mass (*i.e.*, 64 ng) to 10 times the mass resolution (*i.e.*, 3 pg) may be investigated. By varying the platform size, this range can be also varied for larger or smaller samples. Since in our geometry *w_1_* = *l_1_* = *l*(*T*) and *l_2_* = *2w_2_* = *l*, resonance frequency become inversely proportional to *l*(*T*)*^2^*. Considering that both *t*(*T*) and *l*(*T*), subjected to thermal expansion, are proportional to (*1* + *a*(*T* − *T_0_*)), and being the effective mass proportional to (*1* + *a*(*T* − *T_0_*))*^3^*, the resonance frequency results to be inversely proportional to (*1* + *a*(*T* − *T_0_*))*^2^*, where *a* is thermal expansion coefficient. Moreover, both thermal expansion and Young's modulus impact spring constant and effect temperature dependence of the resonance frequency of the resonator. In particular, the spring constant relative change with *T* is given by:

(3) $$\frac{1}{k}\frac{dk}{dT}≅\frac{1}{E}\frac{dE}{dT}+\frac{a}{\left(1+a(T-T_{0})\right)}$$

Since the relative thermal variation of the Young's modulus for silicon nitride is −4.7 × 10^−5^[23], which is larger than *a* = 2.3 × 10^−6^[24], we neglected the size deformation contribution. The spring constant dependence on temperature can be described by the equation [20]:

(4) $$k(T)\approx \left[E_{445}-B(T-T_{445})\right]\frac{t^{3}w_{1}w_{2}}{4\left[w_{2}l_{1}^{3}+w_{1}l_{2}\left(3l_{1}^{3}+3l_{1}l_{2}+l_{2}^{2}\right)\right]}$$

where *T_445_* is 445 K, *E_445_* is the Young's module at *T_445_*, equal to 320.4 GPa for silicon nitride, while *B* (0.0151 GPa/K) is an approximation coefficient. Combining Equations (2),(4), it follows that the resonance frequency dependence on temperature presents a square root trend. Between 300 and 700 K, resonance behavior of a no-load cantilever can be approximated with the equation:

(5) $$f_{0}{\left(T\right)}^{2}-f_{0}{\left(T_{0}\right)}^{2}\approx b(T-T_{0})$$

where *f_0_*(*T*) is the first mode resonance frequency at temperature *T* and *f_0_* is the resonance frequency at the reference temperature *T_0_*. By looking at simulation data, resonator has shown square root temperature dependence, with a coefficient of proportionality *b* equal to −0.8302 K^−1^s^−2^ and a relative frequency variation equal to 0.007. This result does not take into account thermal expansion phenomena that occur on the cantilever paddle rather than on its arm. Equation (5) can be considered accurate in a uniformly heated cantilever, but cannot be used to describe a local heating scenario.

Since areas with high stress level remain close to ambient temperature, a different frequency resonance response was expected and thermal expansion effects on the paddle have been taken under consideration. Looking at Figure 1a, platform expansions along the y-direction are self-compensated while those along x-direction ones shift the center of gravity of the sensor toward its free end, thus decreasing its spring constant. In particular, resonance frequency downs by 0.003% in a rectangular cantilever with the top face of its platform at *T* = 700 K.

Since the resonance frequency increases with sample mass reduction and decreases with temperature, the thermal effects compensate partially the frequency shift due to the mass variation. For example, the resonance frequency shift caused by an increase of mass sample of 0.4 pg is equal to the one caused by the spring constant at a uniform heating temperature of *T* = 700 K. In order to measure the changes of the analyte with temperature, a previous frequency calibration has to be performed.

### 2.2. Details on the Fabrication Process

**Figure 3.**
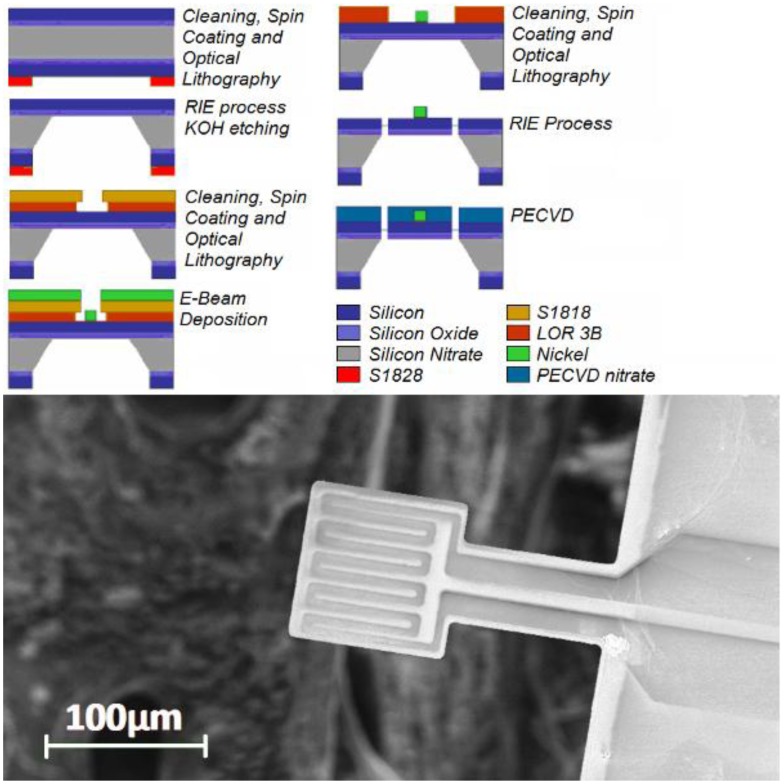
(**a**) NanoTGA Fabrication process; (**b**) SEM top-view sensor image. The MEMS consists of a flexible beam supporting a square platform and of a nickel resistor whose section get smaller on the platform region. The black area is the wafer hole (750 micro × 750 micro) used to locate the material sample on the platform bottom-face.

The fabrication process can be summarized in three main steps: realization of a membrane, deposition of the nickel resistor and fabrication of the cantilever structure. The process flow is sketched in Figure 3a.

The starting material is a silicon wafer with a 2 μm silicon nitride (Si_3_N_4_) layer deposited by low-pressure chemical vapor deposition (LPCVD) on both faces. After acetone and methanol cleaning in ultrasonic bath, the sample is spin coated with 2 μm of positive resist S1828 and baked for 1 min at 120 °C. A 1.5 mm window in the Si_3_N_4_ layer is defined by optical lithography and opened with reactive ion etching (RIE) using O_2_ and CF_4_ (1.5 and 28.5 sccm) at a pressure of 2.5 mbar, with an applied RF power of 100 W and a bias voltage of 250 V. After dry etching, the sample is cleaned with acetone and soaked in a 5 molar of potassium hydroxide solution at 70 °C. After 11 h a freely suspended Si_3_N_4_ membrane, 0.5 mm in size, is obtained. The wafer is cleaned with hot deionized water and acetic acid to remove the salt residuals.

The second step is the fabrication of the thermal resistor. The sample is spin coated with 400 nm of LOR 3B and baked for 3 min at 180 °C. A second layer of 1 μm positive resist S1818 is deposited and baked for 1 min at 120 °C. The resistor geometry is defined by optical lithography, e-beam evaporation of a 30 nm thick nickel layer and lift-off process.

Finally, the cantilever is realized with the same processes used for the membrane. In order to avoid nickel oxidation and ageing, a silicon nitride layer (100 nm) is deposited on the top-face by plasma-enhanced chemical vapor deposition (PECVD). The process involves the use of silane and ammonia (15 and 7 sccm), an RF power applied to the plates of 30 W and a bias voltage of 15 V.

The result is a suspended cantilever (50 × 100 × 2 μm) with a platform (100 × 100 × 2 μm) on the free end, where the nickel resistor cross-section gets smaller (5 × 0.03 μm on the platform and 50 × 0.03 elsewhere). A scanning electron image of complete device is displayed in Figure 3b. The measured resonance frequency of this device was 47 kHz. Arrays with up to 16 devices on 2 × 2 cm wafers were fabricated.

### 2.3. Resistor Calibration

A nickel resistor shaped as a zigzag meander line was used both as heater and as temperature sensor. The thermal dependence of the resistance *R*(*T*) was used to measure the cantilever temperature while the heating efficiency was studied considering thermal dispersion on the platform and thermal dispersion coefficient (*γ*). We evaluated the temperature behavior for *T* < 400 °C and used a second order approximation. For higher temperature higher orders may play a role and further calibration may be required [25,26]. The *R*(*T*) equation is:

(6) $$R(T)=R_{0}\left[1+\alpha \left(T-T_{0}\right)+\beta {\left(T-T_{0}\right)}^{2}\right]$$

where *R*(*T*) is the resistance measured at temperature *T* and *R_0_* is the resistance value at reference temperature *T_0_*. Since only a portion of the resistor is subject to temperature variations, *R*(*T*) is considered as the sum of two contributions: the heater located on the platform (*R^meander^*) and the larger and less resistive interconnecting leads, which we assume to be at constant temperature. Recalling Equation (6), we have:

(7) $$R(T)=R_{0}^{r}+R_{0}^{\text{meander}}\left[1+\alpha \left(T-T_{0}\right)+\beta {\left(T-T_{0}\right)}^{2}\right]$$

The coefficients *α* and *β* obtained from the experiments in furnace, using a four-wire probe measurement set-up, are 1.7 × 10^−3^ K^−1^ and 5.08 × 10^−6^ K^−2^ respectively. The sensor has been tested for temperature below 450 °C but the system, with a suitable heater design, may support operating temperatures above 1,000 °C. In the range under observation the estimated error in temperature measurements has been quantified to be ±2.5°.

When the nickel resistor is used to increase the temperature, the power dissipated by Joule effect increases the temperature of the sensor according to following equation:

(8) $$dT=T-T_{0}=\gamma VI$$

The coefficient *γ* approximates the overall response of the system, including radiative dispersion in vacuum. It results equal to 5 × 10^5^ K/J and confirms the presence of a large temperature gradient and the localization of the thermal phenomena on the platform area.

## 3. NanoTGA Implementation

The high performance of our microelectromechanical system has been tested by optical setup and dedicated electronic system, which are able to detect sub-nm mechanical deflections and are suited to determine the cantilever's vibrational amplitude.

### 3.1. Experimental Setup

**Figure 4.**
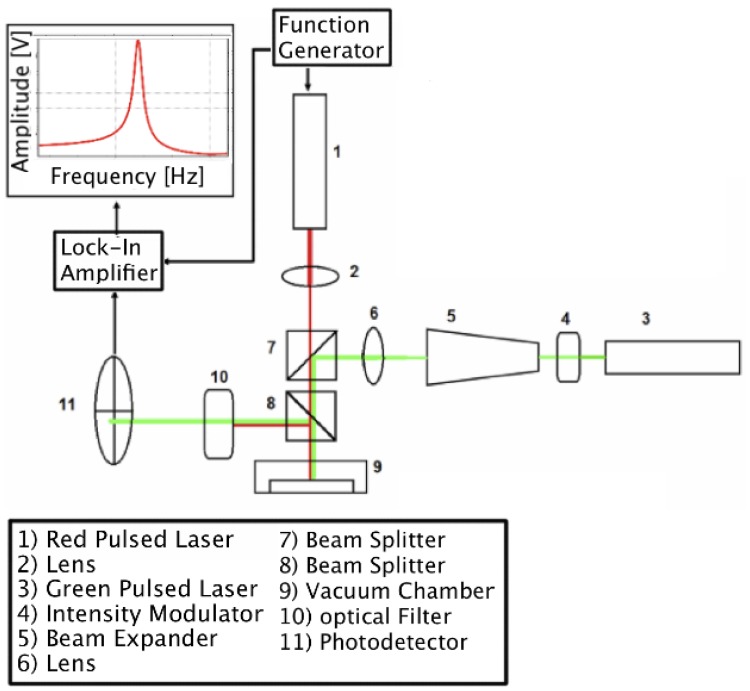
Scheme of the electronic set-up used for the thermogravimetric analysis. The sensor is placed in a vacuum chamber and a pulsed focalized red laser excites cantilever vibrations. A green laser and photodiode are used to read out the cantilever deflections and this information is converted by a lock-in amplifier into a resonance curve.

The resonance frequency of the cantilever and its temperature dependence have been measured by optical deflection method under vacuum. The dedicated opto-electronic system scheme is shown in Figure 4.

The sensor is placed and locked in a MACOR holder under controlled pressure of 1 × 10^−3^ mbar; a frequency modulated red laser is focused on a spot of few microns on cantilever's base with a lens having focal length of 50 mm and is used to actuate the cantilever. A continuous wave green laser (DPSS @ 532 nm) focused on the platform by means of a beam expander and a lens (focal length of 50 mm) is used to read the cantilever displacements. The reflected lights pass through a band pass optical filter (CWL from 150 to 550 nm) to isolate the green component, which is detected by a fast four-quadrant photo-diode (Hamamatsu S7379-01, cut off frequency 80 MHz). A lock-in amplifier (Stanford Research Systems SR830) elaborates the response of the photodiode in reference with the sinusoidal signal that modulates the red laser. The result is the resonance curve of the cantilever.

The temperature is tuned by feeding the resistor with a 150 kHz alternate voltage of variable amplitude. A DC voltage for the same purpose cannot be used, since a DC electric field bends piezoelectrically the cantilever and, at high voltage, the optical path is driven out of the sensitive area of the detector. The heater is feedback looped with the temperature real-time four-wires measurement.

Dedicated control software has been developed within the Labview platform. In detail, two frequency scans, before sample deposition and after thermal test, provide the total sample weight before the analysis and the residual mass after analysis.

Information on sample status are given every time a desire environment change happens, such as a modification of the sample mass or a temperature increase. The resonance frequency shift provides the information about the sample mass changes. By reference to Equation (2), the temperature dependence of the resonance frequency can be expressed as:

(9) $$\frac{1}{f}\frac{df}{dT}=\frac{1}{2k}\frac{dk}{dT}-\frac{1}{2\left(m_{\text{eff}}-m_{S}\right)}\frac{dm_{S}}{dT}$$

The variation in the mass of the sample can then be calculated using:

(10) $$m_{S}=m_{\text{eff}}\left(\frac{f_{S}{\left(T\right)}^{2}}{f{\left(T\right)}^{2}}-1\right)$$

where *f_S_* is the resonance frequency of the no-load cantilever and *f* is the resonance frequency of the loaded cantilever, both considered with their temperature dependence.

### 3.2. Material and Measurement of the Mass Changes

Water liquid-vapor phase transition is used as a first calibration test of the system. A second test with several distinct transitions is provided by a single polyurethane microcapsule filled with chlorobenzene.

#### Synthesis Microcapsules

The capsules were obtained by interfacial polymerization [27]. Briefly, urethane prepolymer was synthesized by mixing tolylene2,4-diisocyanate (21.85 g) and cyclohexanone (141.65 g) in a flask. Then we added 1,4-butanediol (4.12 g, 46 mmol) to the mixture under agitation at 300 rpm and left the solution for 24 h at 80 °C. After evaporation of the cyclohexanone under reduced pressure at 100 °C, a yellowish and viscous prepolymer was obtained. We mixed a portion of the synthesized urethane prepolymer (2.91 g) with chlorobenzene (12.5 g) and, after dissolution of the prepolymer in the solvent, we first added to the mixture a 15 wt % gun arabic aqueous solution (28.0 g) and then a ethylene glycol (1.5 g, 24 mmol) at 50 °C. After 2 h of heating at 70 °C with stirring at 2,000 rpm, the solvent-encapsulated microcapsules were synthesized. As a result of the synthesis, microcapsules of diameter ranging between 30 and 90 μm, in agreement with the size of the MEMS platform, were obtained. In order to operate with few capsules, ideally single capsules on each cantilever, the solution have been properly diluted by a factor 140 by adding Milli-Q water (1 mL) to the original solution (70 μL).

## 4. Results and Discussion

We compensated temperature effects on frequency shift by recording resonance frequency as a function of temperature of an unloaded cantilever. The performance of the nanoTGA system was then tested using deionized water and microcapsules samples.

### 4.1. Unloaded Cantilever

Frequency variation of the unloaded sensor has been first recorded at room temperature. In this way, the resonance changes due to the spring constant temperature dependence were evaluated.In order to remove all undesirable water and absorbed contaminants from the cantilever platform, where the sample has to be placed, the nanoTGA has been heated up to 300 °C using the integrated resistor and then cooled down before the measurement.

**Figure 5.**
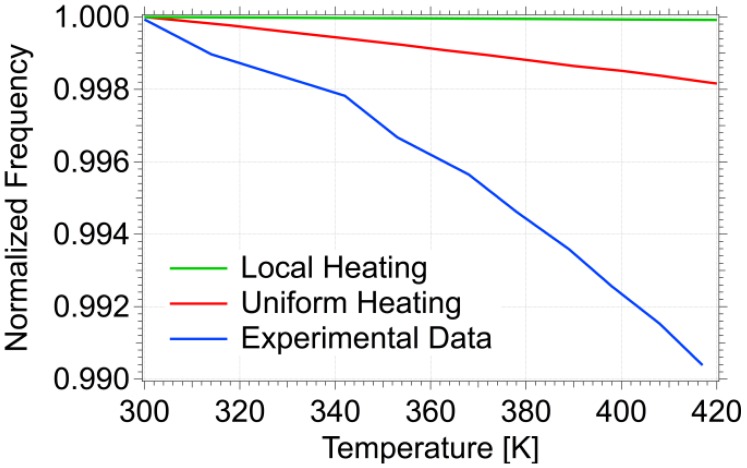
Sensor calibration of the unloaded cantilever: shift in resonance frequency as a function of the temperature. The red and green lines show the results obtained by mathematical simulation for uniform and local heating conditions, respectively, while the blue one shows the experimental data.

Figure 5 shows a comparison between the results obtained by mathematical simulation and those obtained in the real temperature scan. Both curves can be approximated by Equation (5). Although the experimental curve confirms qualitatively the numerical simulations, the decrease of resonance frequency with increasing temperature is higher than expected, being the coefficient of proportionality between *f_0_*(*T*) and *dT* ‒1.6 K^−1^s^−1^. Over the temperature range reported in Figure 4, the mass sensitivity is 10 fg/°C, significantly below the mass of a single polyurethane microcapsule [27]. A possible explanation of the difference observed between experimental and simulated thermal response is as follows. When performing Comsol simulations we did not take into account the nickel heater thermal expansion (1.3 × 10^−5^ K^−1^), which is indeed one order of magnitude larger than nitride one. As a consequence, at increasing temperatures the Si_3_N_4_/nickel bilayer bends with increasing thermal stress, which induce plastic deformation of sensor structure and make resonator temperature dependence more important than expected.

### 4.2. Milli-Q Water

A first advantage of the nanoTGA approach is that it's extremely low thermal inertia allows precise measurements of phase transitions. We demonstrated this issue by measuring the evaporation of droplet of water. In order to deposit the amount of Milli-Q water (less than 600 μm^3^) on the platform surface, the sensor was placed in contact with a water droplet (1 μL). By loading the chip in a vacuum chamber and measuring the resonance frequency before and after the sample deposition, the presence of a nanogram droplet on the cantilever was verified.

**Figure 6.**
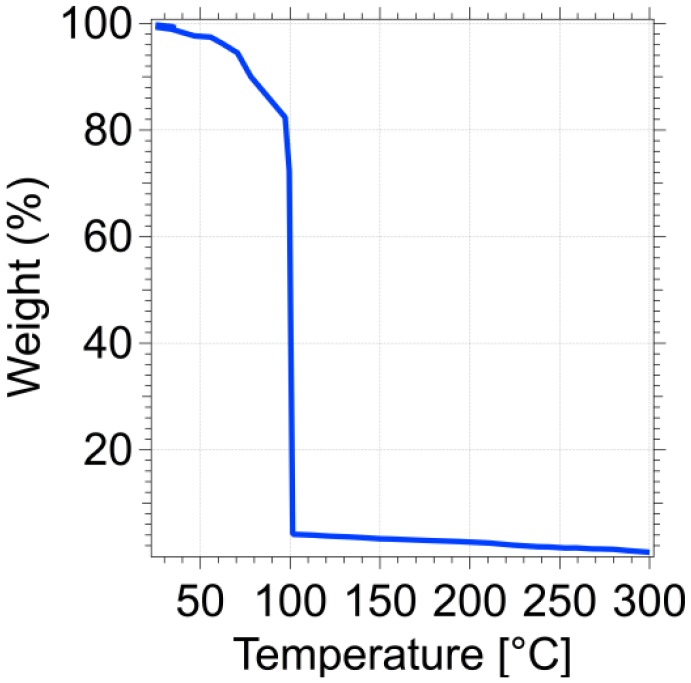
Water-loaded cantilever. Variation in the percentage weight of the water drop as a function of the temperature.

The temperature is raised from 50 to 300 °C (heating rate: 5 °C/min) with steps of 10 °C, that correspond to a resistance variation of 50 Ω. The mass *versus* temperature curve is corrected taking into account the intrinsic temperature dependence on the resonance frequency. The actual mass variation is displayed in Figure 6. The curve shows an abrupt change of mass at 100 ± 3 °C. At this temperature about 78% of the loaded water mass evaporates. The water mass changes from 4.6 ng before the process to 1 ng after the process and the weight loss profile *vs.* temperature is much sharper than those obtained with commercial systems [28]. Because of the extremely low thermal capacity of the cantilever (about 10^−9^ J/K), comparable to that of the sample, the whole system does not increase temperature at the liquid/vapor water phase transition. In other words, our approach allows a direct measurement of the sample temperature. Commercial systems, on the other hand, measure the temperature of furnaces with thermal capacity much larger than the sample. As a consequence, the measured temperature may differ from sample temperature, especially if a temperature ramp is performed. This results in a significant broadening of the TGA response that can span over 100 °C.

### 4.3. MicroCapsule

A second advantage of the nanoTGA approach is that it allows one to separate the contribution of each element, thus the characterization of poly-dispersed materials, while commercial systems provide only averaged information. To this purpose we performed TGA on polyurethane microcapsules. The permeability of the individual capsule is a function of shell thickness and uniformity. This parameter is connected with the initial capsule radius and it affects the sample temperature curve; therefore, the rupture temperature decreases as the microcapsule permeability increases.

**Figure 7.**
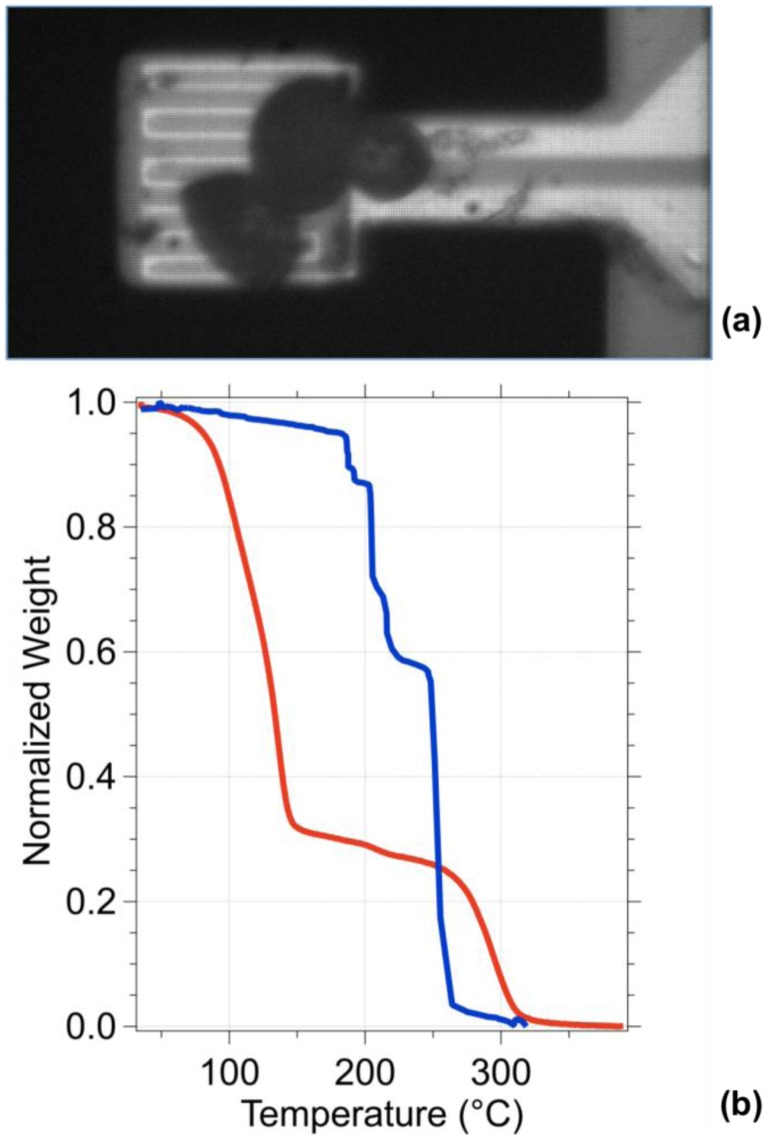
Microcapsule loaded cantilever. (**a**) Loaded sensor image. The picture has been taken with a CCD camera and an optical microscope. The dark-grey circles on the cantilever free end are three microcapsules presented in the solution; (**b**) Comparison between temperature analysis data measured using a commercial TGA (red) and cantilever-based TGA (blue).

Figure 7a shows a picture taken with an optical DIC microscope of a nanoTGA with three microcapsules on its platform. The result obtained by analyzing this group of microcapsules by our nanoTGA is shown in Figure 7b (blue curve), where we can recognize the rupture temperature of each capsule and the degradation of all the shells. The rupture temperatures were 138, 157 and 175 °C, where we can observe the gradual evaporation of the chlorobenzene inside the shells.

The total sample mass deposited on the nanoTGA was 3.3 ng with an individual capsule weight of 1.1 ng. Upon increasing the annealing temperature individual capsules break releasing their chlorobenzene content while all polyurethane shells sublimate together at 250 °C, with a sharp transition.

While the commercial TGA can measure only the average weight loss of a large number of capsules characterized by a broad range of rupture temperature [29], nanoTGA can detect the mass change of a single microcapsule thus providing a significant correlation between size and thermal properties [30,31]. Indeed, the MEMS device provides a more accurate analysis of rupture temperature and thermal degradation temperature of the polyurethane shell.

In Figure 7b the normalized percentage weight loss of microcapsule agglomeration measured performing the TG analysis with a commercial set up (red) is also presented. The commercial test has been performed on a 6.6 mg microcapsule solution in a nitrogen-filled chamber and the sample behavior was observed between 35 and 350 °C with temperature ramp of 20 °C/min (blue). The blue curve presents a smooth weight transition around the rupture temperature between 100 and 150 °C, while the thermal degradation of the polyurethane shell is visible beyond 250 °C.

## 5. Conclusion

We have proposed a cantilever-based nanoTGA, which integrates both the heater and the temperature sensor within the cantilever. The frequency and temperature responses were optimized by FEM analysis and experimental calibration. The devices showed a fast thermal response with virtually no intrinsic thermal capacity and small mass sensitivity. In particular, single particle thermal analysis was demonstrated.
